# NMR Metabolomics in Serum Fingerprinting of Schizophrenia Patients in a Serbian Cohort

**DOI:** 10.3390/metabo12080707

**Published:** 2022-07-29

**Authors:** Katarina Simić, Nina Todorović, Snežana Trifunović, Zoran Miladinović, Aleksandra Gavrilović, Silvana Jovanović, Nataša Avramović, Dejan Gođevac, Ljubodrag Vujisić, Vele Tešević, Ljubica Tasić, Boris Mandić

**Affiliations:** 1 Institute of Chemistry, Technology and Metallurgy, National Institute, University of Belgrade, Studentski trg 12-16, 11000 Belgrade, Serbia; katarina.simic@ihtm.bg.ac.rs (K.S.); ninat@chem.bg.ac.rs (N.T.); dgodjev@chem.bg.ac.rs (D.G.); 2 University of Belgrade-Faculty of Chemistry, Studentski trg 12-16, 11000 Belgrade, Serbia; snezanat@chem.bg.ac.rs (S.T.); ljubaw@chem.bg.ac.rs (L.V.); vtesevic@chem.bg.ac.rs (V.T.); 3 Institute of General and Physical Chemistry, Studentski trg 12-16, 11158 Belgrade, Serbia; zmiladinovic@iofh.bg.ac.rs; 4 Special Hospital for Psychiatric Diseases “Kovin”, Cara Lazara 253, 26220 Kovin, Serbia; gavrilovicaleksandra74@gmail.com (A.G.); silvana.jovanovic555@gmail.com (S.J.); 5 Institute of Medical Chemistry, Faculty of Medicine, University of Belgrade, 11000 Belgrade, Serbia; natasa.avramovic@med.bg.ac.rs; 6 Institute of Chemistry, Organic Chemistry Department, State University of Campinas, Campinas 13083-970, SP, Brazil; ljubica@unicamp.br

**Keywords:** schizophrenia, metabolomics, biomarkers, NMR, chemometrics, serum metabolites

## Abstract

Schizophrenia is a widespread mental disorder that leads to significant functional impairments and premature death. The state of the art indicates gaps in the understanding and diagnosis of this disease, but also the need for personalized and precise approaches to patients through customized medical treatment and reliable monitoring of treatment response. In order to fulfill existing gaps, the establishment of a universal set of disorder biomarkers is a necessary step. Metabolomic investigations of serum samples of Serbian patients with schizophrenia (51) and healthy controls (39), based on NMR analyses associated with chemometrics, led to the identification of 26 metabolites/biomarkers for this disorder. Principal component analysis (PCA) and orthogonal partial least squares discriminant analysis (OPLS-DA) models with prediction accuracies of 0.9718 and higher were accomplished during chemometric analysis. The established biomarker set includes aspartate/aspartic acid, lysine, 2-hydroxybutyric acid, and acylglycerols, which are identified for the first time in schizophrenia serum samples by NMR experiments. The other 22 identified metabolites in the Serbian samples are in accordance with the previously established NMR-based serum biomarker sets of Brazilian and/or Chinese patient samples. Thirteen metabolites (lactate/lactic acid, threonine, leucine, isoleucine, valine, glutamine, asparagine, alanine, gamma-aminobutyric acid, choline, glucose, glycine and tyrosine) that are common for three different ethnic and geographic origins (Serbia, Brazil and China) could be a good start point for the setup of a universal NMR serum biomarker set for schizophrenia.

## 1. Introduction

Schizophrenia is a widespread mental illness ranked in the top 25 causes of disability worldwide [1], which leads to significant functional impairments and premature death. The World Health Organization (WHO) estimated that schizophrenia affects 20 million people worldwide [2,3]. Global total costs for this illness are difficult to calculate because of their complex and various influences on society and the economy. Only in the USA, yearly costs are estimated at over 100 billion dollars. The economic burden of schizophrenia varies from 0.02% to 1.65% of the gross domestic product in low, medium, and high-income countries, with an indirect cost contribution of 50–85% [4,5,6].

The state of the art in this research area indicates that this mental illness is the result of complex interactions between genetic and environmental factors, and the underlying pathophysiology is not completely understood. The current diagnostic criteria for psychiatric diagnosis are based on clinical phenomenology, and they are limited to psychiatrist judgment after a standard clinical interview and reports from patients or caretakers. Diagnosis is significantly hampered in cases with advanced mental disorders due to difficult communication with the patient and/or lack of credible information from the environment. Besides gaps in the understanding and diagnosis of the illness, there is also a need for personalized and precise approaches to patients through customized medical treatment and reliable monitoring of treatment response [7]. In order to fulfill the existing gaps, the establishment of a universal set of disorder biomarkers is a necessary step.

NMR spectroscopy and MS spectrometry are the two most-used platforms in metabolomics. Compared with MS analyses, NMR is less sensitive and has limited resolution. However, NMR analyses offer high reproducibility and quantitative accuracy using intact biospecimens without the need for separation [8]. Previous metabolomic and lipidomic analyses of fluid (blood and urine) samples of patients with schizophrenia [9,10,11,12,13,14,15,16,17,18,19,20,21,22,23,24,25,26] led to the detection of metabolites as potential biomarkers. NMR-based metabolomics of patient serum samples offered 59 potential biomarkers for schizophrenia [9,10,11,12,13,14,15]. However, due to the lack of comparative investigations of patient samples with different geographical and ethnical origins, supported by an adequate systematic methodology, a universal set of biomarkers (fingerprints of illness) has not been established.

In accordance with the objective of the “Mental Health Action Plan 2013–2030” created by the WHO for strengthened information systems, evidence, and research [27], and in order to support efforts for the establishment of the universal fingerprint for schizophrenia, we performed a metabolomic investigation of a cohort of Serbian schizophrenia patients serum samples based on NMR analyses associated with chemometrics.

## 2. Results

This study included 51 patients with schizophrenia and 39 healthy individuals in the control group, and the two groups of the investigated individuals were carefully paired regarding sex and age. Schizophrenia patients underwent the same treatment with the antipsychotics regarding dose and time, illness stage, and symptoms before hospitalization. NMR analyses of blood samples were performed in triplicate.

### 2.1. Chemometrics

The ^1^H-NMR data sets were processed applying Bruker Topspin software, and the spectra phases and baselines were corrected using automatic options. The 0th order phase correction was carried out manually, contributing to noise variance removal (Supplementary Material, Figure S1). Finally, the data set was processed by GNAT software and analyzed by chemometrics.

#### 2.1.1. Exploratory Analysis

The most common method for exploratory data analysis is principal component analysis (PCA). When the number of variables is 32 K, as in this case, the simple univariate methods are not easily applicable. Nevertheless, univariate statistics such as skewness and kurtosis could be informative to some extent and helpful in determining the method of scaling or variables regions with significant discrepancy from a normal distribution or even potential outliers. Kurtosis for a normally distributed data set should be near 3. Pronounced positive values for both statistics were observed in the region of spectra between 3.2 to 3.9 ppm as well as at 1.21 ppm. Therefore, samples that contributed to this area of spectra are considered possible outliers. Spectral regions below 0 ppm and above 8 ppm, without the presence of any significant resonance signals, were also removed from further consideration. After careful analysis of the statistics, 6 samples (i.e., triplicates of two ‘Schizophrenia’ patients) were identified as potential outliers (Supplementary Material, Figure S2). Additional two outliers were identified in PCA analysis. The reason for omitting 8 spectra from the data was inadequate water resonance suppression or high dilution of the samples, which provided a very low signal-to-noise ratio. These outliers were, therefore, removed from the data set for the rest of the study.

#### 2.1.2. PCA Models

**Figure 1:**
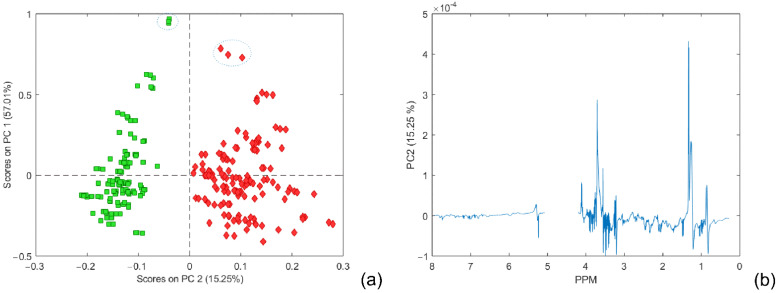
(**a**) PCA score plots of the first two components. The schizophrenia cohort is shown in red, and the control group in green. (**b**) PC2 back-scaled projection of loading coefficients. The empty part of the loading plot belongs to the water resonances region.

In order to obtain the most reliable PCA models, different centering and scaling methods were used: Pareto centering and scaling, autoscaling, and class centroid centering and scaling. The resulting number of PC components of a PCA model was determined using RMSECV from 7-fold cross-validation (described in the experimental section). The PCA model using Pareto scaling with mean centering data accounted for a total variance of 90.95%, and the first two components provided a very good separation between the two main classes (Figure 1). Other results are illustrated in the Supplementary Material (Figures S3 and S4).

The most positive contribution to the PC2 loading graph (Figure 1b), corresponding to the class ‘Schizophrenia’, could be identified around 1.33 ppm (doublet: 1.32 ppm; 1.34 ppm) and around 4.11 ppm (quartet: 4.09 ppm; 4.10 ppm; 4.12 ppm; 4.13 ppm), which could be assigned to the signals of lactate, and in the area between 3.71 to 3.61 ppm typical for sugar molecules. Additionally, in PC2 loading, the corresponding variables at 0.84 ppm, 3.21 ppm, 3.55 ppm, and 5.28 ppm show the characteristic dispersion-phase pattern of chemical shift variation where the shape is similar to the first-derivative curve of a peak. Both positive and negative parts of the peak intensity are approximately equivalent. Resonances at these positions are mostly related to broad signals, which could be attributed to peak position variation and lead to discrimination [28]. The most pronounced signals in mean-centered NMR spectra were positioned at 0.88, 1.28, 1.58, 2.04, 2.24, 2.75, and 5.31 ppm. Those were in good agreement with the loading coefficients of variables that contributed to the higher score projection to the PC 1 component. It could be seen that all scores with higher positive projection to the PC 1 component (rounded with blue ellipses in Figure 1a) have significantly higher intensity contribution in mean-centered PC 1 loadings; nevertheless, they cannot be considered outliers.

#### 2.1.3. OPLS-DA Models

Pattern recognition data analysis was carried out in two steps. In the first stage, all the variables were mean-centered (or class centroid centered); thereafter, they were autoscaled by dividing each variable by its standard deviation (or pooled standard deviation) and then analyzed using a supervised pattern recognition method—orthogonal projection on latent structure (O-PLS), which was developed by Trygg et al. [29]. The O-PLS model represents a modification of the PLS model, which separates the systematic variation on X into three parts, the first one that is linearly related to Y, the second part that is orthogonal to Y (structured noise), and the last one contains the residual variance [30,31].

**Figure 2:**
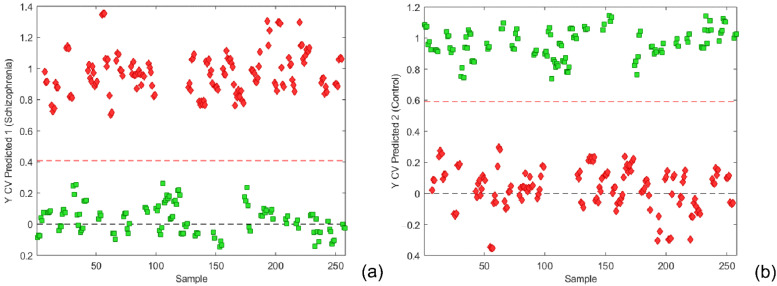
(**a**) Y CV Predicted for the class ‘Schizophrenia’ and threshold value of 0.4086; (**b**) Y CV Predicted for the class ‘Control’ and threshold value of 0.5914 using autoscaling. The schizophrenia cohort is shown in red, and the control group in green.

Prediction capabilities were tested for the chosen number of components with independent test data set comprising 32 samples of ‘Schizophrenia’ and 39 samples belonging to the ‘Control’ class, a total of 71 samples. As a result, the final number of components was selected according to the minimum value of the root-mean-square error of prediction (RMSEP) obtained for a different number of model components. Predictions for both classes using autoscaling as a preprocessing method for centering and scaling are presented in Figure 2. The classification threshold for each class model is calculated using the Bayesian method [32,33]. For the ‘Schizophrenia’ and ‘Control’ classes, the thresholds were determined as 0.4086 and 0.5914, respectively.

The corresponding confusion matrix for the classification of the external test dataset according to the PLS-DA model with autoscaling preprocessing is shown in Table 1. It could be seen that, with 2 misclassified samples from ‘Schizophrenia’ (with an accuracy of 0.9718) for the independent test dataset, the model has satisfied prediction capability (Supplementary Material, Figure S5).

**Figure 3:**
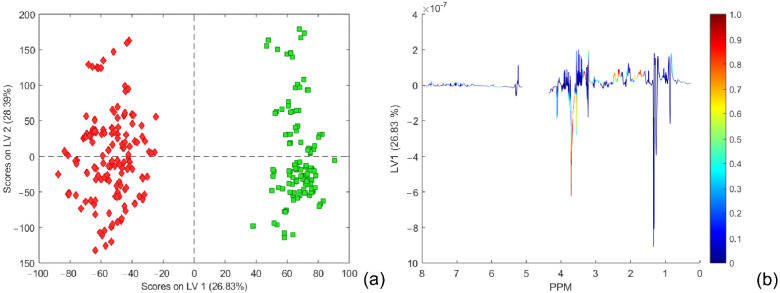
(**a**) Score plots of the first two LV components of the OPLS DA model using mean-centering and unit variance scaling (for a 4-component model, RMSEC = 0.0934 and RMSECV = 0.1304). The schizophrenia cohort is shown in red, and the control group in green. (**b**) Back-scale projection of loading vector LV 1 to coloring coded according to the absolute value of the particular loading weighted by correlation of the spectral data set and score matrix from the OPLS-DA model. Part of the loading plot belonging to the residual water signals was omitted from the plot.

The score plot of the first predictive LV 1 component (comprising 26.83% of the variance) to the first orthogonal LV 2 component (comprising 28.39% of the variance) is shown in Figure 3. The total variance covered by the OPLS-DA model was 72.57% for the X block of the dataset. The back-scale projection of the predictive component is given with color coding according to the loading correlation proposed by Wiklund et al. [30], also named an S line plot [34].

#### 2.1.4. PLS-DA for Unequal Class Size

**Figure 4:**
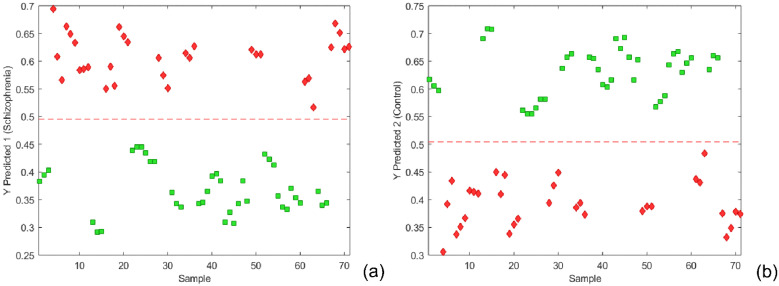
(**a**) Y predicted for the class ‘Schizophrenia’. (**b**) Y predicted for the class ‘Control’ from an external test dataset using class centroid centering and scaling. The schizophrenia cohort is shown in red, and the control group in green.

**Figure 5:**
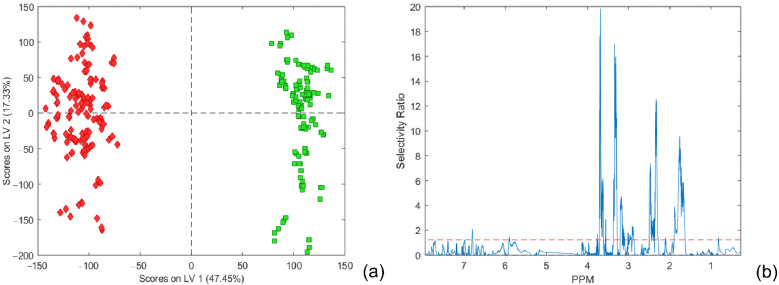
(**a**) Score plot of the first predictive LV 1 and the first orthogonal LV 2 components (for a 4-component model, RMSEC = 0.0845 and RMSECV = 0.1071). The schizophrenia cohort is shown in red, and the control group in green. (**b**) Selectivity ratio plot.

When the number of samples in the analyzed groups is unequal or unbalanced, then using a standard approach in data pretreatment for PLS-DA analysis will not usually result in the most appropriate class separation boundary [35]. In such a case, the overall mean centering shifts the center of gravity towards the larger class group, resulting in the shifting of class separation boundary towards the larger class group, producing in this way more misclassified samples from this class. To overcome this problem, the method of weight centering the X data matrix for PLS by subtracting the average of the means of the two-class groups from the columns was proposed. Accordingly, the center of gravity became the same for X. In addition, pulled standard deviation for both classes could also be incorporated in the modified centering and scaling, so-called class centroid centering and scaling. For centering and scaling, we have used both autoscaling (mean centering and unit variance scaling) and class centroid centering and scaling. In such a way, we were able to compare the influence of both methods on PLS-DA model performance and class member predictability. The results are shown in Figure 4 and Figure S5. As illustrated in Figure 4, better prediction capabilities were observed for the model constructed from class centroid centering and scaling data. The separation between classes, in this case, was very good, without any misclassified samples. Apparently, the higher variance explained by the predictive component (comprising 47.45% of the total variance, Figure 5a) accomplishes better separation between classes and decreases the threshold (Figure 4) when compared with the OPLS-DA model with autoscaling preprocessing (Figure S5). In order to confirm that obtained OPLS-DA models are not over-fitted, a permutation test was performed (within PLSToolbox) using 200 iterations for each model. Obtained results indicate that original models are more unlikely to be over-fitted, and their test results for each of the classes are presented in Supplementary Materials, Figure S6.

#### 2.1.5. Discriminatory Metabolites and Variable Importance in Projection Signatures

There are many ways to select discriminating variables with diagnostic values, such as variables with large regression coefficients [36], normalized covariance (PLS weights), [28,31] between spectral variables and the response, variable importance in projection (VIP) [34,37], and selectivity ratio plots [38]. The covariances between the response Y and the spectral variables (usually labeled by w1 and called weights in standard PLS) have also been proposed for variable selection. Cloarec et al. [28,31], in their work, slightly broadened and adopted this idea. Particularly, in the case of OPLS-DA models based on autoscaled data with two classes, the value of loading for a variable corresponds to the correlation coefficient between the variable and the class descriptor. They introduced so-called back-scale projection loading, which is plotted using for each point a color corresponding to the weight value in the model that represents the correlation of the X variable with Y. In this way, color-coding could be used for weighting coefficients to distinguish the relative importance of variables. However, a more robust and sensitive indication in this sense was approved, at least in this work, by the selectivity ratio plot proposed by Rajalahti et al. [38]. As can be seen from Figure 3 and Figure 5, both methods indicate almost identical ranges of chemical shifts inside NMR spectra as potential biomarker assignation areas for the distinction between two classes of samples: ‘Schizophrenia’ and ‘Control’. The most intensive difference could be recognized in the range of 3.61–3.71 ppm belonging to sugar molecules, and according to the loading plot, it is more relevant to the class ‘Schizophrenia’ than for the ‘Control’ class. The spectral parts in the ranges of 1.63–1.89 ppm, 2.29–2.49 ppm, and 3.28–3.35 ppm could be clearly identified from the selectivity ratio plot and connected to the class ‘Control’ as more significant for class separation. Chemical shifts ranging from 3.14 to 3.21 showed slightly lower importance than previously mentioned but should also be considered as potentially important biomarkers. It should be noticed that both methods, as well as VIP (data not presented), reveal almost identical variables; nevertheless, the selectivity ratio provided a more sensitive method for variable selection from the OPLS-DA model in this work.

### 2.2. NMR Analyses

**Figure 6:**
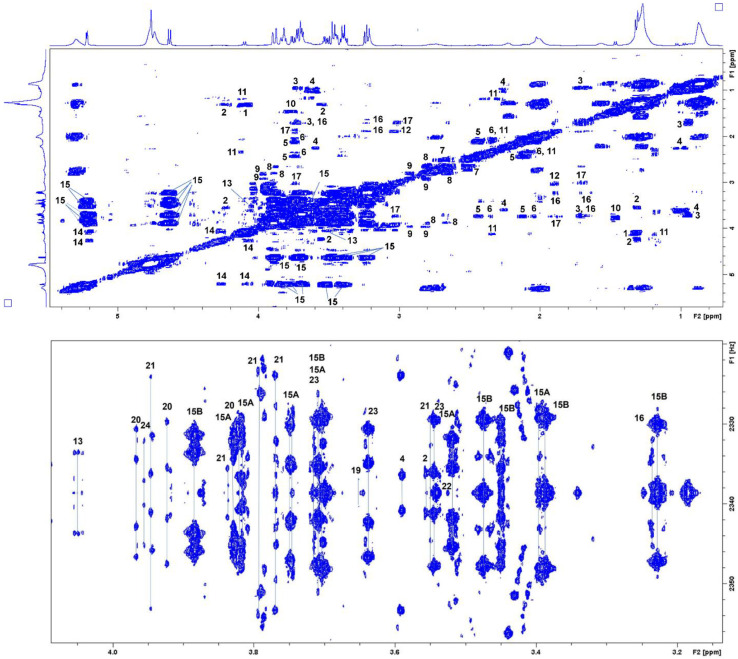
TOCSY ((**upper**) panel) and 2DJ ((**lower**) panel) spectra of one sample from the Serbian cohort of schizophrenia patients in 0.7–5.5 ppm and 3.1–4.1 ppm, respectively.

In accordance with the results of chemometric analyses, the identification of metabolites as potential biomarkers in blood samples of patients with schizophrenia from a Serbian cohort was performed based on analyses of spectral 2D NMR data obtained in TOCSY, 2DJ, and HSQC experiments. TOCSY spectral data (Figure 6) led to the identification of 20 metabolites, while 25 metabolites were identified based on 2DJ experiments. HSQC analyses confirmed the presence of 14 metabolites in schizophrenia patients’ serum samples. In total, we established a set of 26 metabolites as serum biomarkers for schizophrenia. The set of identified metabolites/biomarkers with spectral data is presented in Table 2.

## 3. Discussion

Metabolomic analyses of serum samples of Serbian patients with schizophrenia and healthy volunteers as a control group led to the identification of 26 metabolites as a biomarker set for this psychiatric illness. Aspartate/aspartic acid, lysine, 2-hydroxybutyric acid, and acylglycerols were identified for the first time in the patients’ serum samples as schizophrenia biomarkers based on NMR-metabonomics. Changes in these biomarkers could be a consequence of bioenergetic abnormalities in schizophrenia patients. The lack of energy is now recognized [10,11], metabolic pathways are changed, the tricarboxylic acid cycle is inhibited, and glycolysis is enhanced. For supplying energy, fatty acid beta-oxidation is stimulated. Therefore, acylglycerols (biomarker established in this paper) are mobilized, and they can be used by the tissues as an energy source. During the prolonged absence of sufficient amounts of glucose and lack of oxaloacetate (due to gluconeogenesis), ketone bodies become an energy source. Biomarker 3-hydroxybutyric acid may point to the formation of ketone bodies in schizophrenia. Acetoacetate formed is then reduced to 3-hydroxybutyric acid. On the other hand, 2-hydroxybutyric acid (also an identified biomarker in this study) derives from alpha-ketobutyrate. It is produced by amino acid catabolism (threonine and methionine) and glutathione anabolism (cysteine formation pathway). It has been shown that 2-hydroxybutyric acid generally appears in situations related to deficient energy metabolism and impaired glucose regulation that appears to arise due to increased lipid oxidation and oxidative stress [39].

It is interesting that fifteen of the twenty standard amino acids that are commonly found in proteins have been identified as biomarkers in schizophrenia patients’ serum samples. Previously detected amino acid biomarkers such as alanine and glutamate may suggest the possible disturbed use of glucogenic amino acids via degradation and deamination processes [12]. The carbon skeletons of the glucogenic amino acids, which are degraded to pyruvate or citric acid cycle intermediates, can subsequently be used in gluconeogenesis. Polar lysine and aspartate/aspartic acid are established for the first time by NMR experiments. The excess glutamate (resulting from enhanced deamination of amino acids) could be turned into the TCA cycle, and it could disrupt the balance of alanine, aspartate and glutamate metabolism [40]. The alterations of mentioned metabolism pathways would aggravate the neurological damage.

The other 22 biomarkers were previously identified in serum samples of patients from Brazil and China [9,10,11,12,13,14,15]. Tasic et al. [9,12] established a set of 30 biomarkers based on 1D and 2D NMR analyses (CPMG, HSQC, and HMBC) of a Brazilian cohort of schizophrenia patients’ serum samples. Liu et al. [10] analyzed serum samples by CPMG, NOESY, and 2DJ NMR experiments, while Wang et al. [11] identified biomarkers by CPMG experiments. Jointly, they offer 44 NMR-based serum biomarkers for schizophrenia patients from a cohort from China (Table 3).

Established NMR-based serum biomarker sets of Serbian, Brazilian, and Chinese schizophrenia patients overlap in 13 metabolites. Comparing just Serbian and Brazilian results, the biomarker sets show an overlap of 18 metabolites, while Serbian and Chinese sets overlap with 17 biomarkers, and Brazilian and Chinese biomarker sets have 15 mutual metabolites. On the other hand, each of these NMR biomarker sets contains metabolites that have not been identified in the serum samples of patients of different geographic and ethnic origins. Of course, the differences in the offered NMR biomarker sets could be mainly caused due to lack of consistency in the strategy and methodology in the analysis of samples with different origins. Nevertheless, thirteen metabolites (lactate/lactic acid, threonine, leucine, isoleucine, valine, glutamine, asparagine, alanine, gamma-aminobutyric acid, choline, glucose, glycine, and tyrosine), identified by various NMR experiments and different instruments in all three biomarker sets and in the serum samples from different origins, could be a good starting point for further efforts in order to establish a universal NMR serum biomarker set for schizophrenia.

## 4. Materials and Methods

### 4.1. Sampling and Sample Preparation

Sampling was performed in compliance with the ethics committee approval of the Special Hospital for Psychiatric Diseases “Kovin”, University of Belgrade—Faculty of Chemistry and Blood Transfusion Institute of Serbia. Blood samples of selected medically treated patients with schizophrenia were provided from the Special Hospital for Psychiatric Diseases “Kovin,” and corresponding samples of healthy controls were provided from the Blood Transfusion Institute. In compliance with ethics committee approval, patients or their caretakers and healthy volunteers signed written consent for the donation of their blood samples for this research. Through this research, 51 blood samples of male (25) and female (26) patients, 32 to 68 years old, were analyzed. A total of 15 patients were using antipsychotics of the first generation (flufenazin, hloropromazin, haloperidol, levomepromazin), 20 patients were using antipsychotics of the second generation (clozapin, risperidon, aripiprazol, kvetiapin, olanzapin), 7 patients were using antipsychotics of the first generation and second generation and 9 patients were using anxiolytics (clonazepam, lorazepam, diazepam, pregabalin). The control group consisted of 39 healthy volunteers, males (27) and females (12), 23 to 60 years old. Sample preparation counted on three independent blood samples. After sample collection, blood was kept on ice for one hour and centrifuged. Obtained serums were stored at −80 °C. Prior to NMR analyses, serum samples were diluted with D_2_O (vol., 1:1).

### 4.2. Chemometrics

#### 4.2.1. Software

All data processing in this work was applied using toolboxes and software implementations, including in-house developed scripts/codes conducted under MATLAB version 9.7 (MathWorks Natick, Massachusetts, USA) [41]. Preprocessing and chemometrics analysis of ^1^H-NMR spectral data were performed by PLS Toolbox version 8.9.1 [32]. Reading in ^1^H-NMR spectra into MATLAB workspace was accomplished by General NMR Analysis Toolbox (GNAT) version 1.2 [42], and in some instances, by predeveloped macros from matNMR version 3.9.144 [43]. Alignment of specific spectral regions inside the ^1^H-NMR spectra was implemented through Interval Correlation Optimized shifting (icoshift) version 3.0 beta [44].

#### 4.2.2. Reading in Data

To read in data into MATLAB workspace for further chemometric analysis, the matNMR script matNMRReadBrukerSpectra was exploited as a part of an in-house routine developed in order to automate this process. In this way, the ^1^H-NMR spectral dataset was set, preserving the original spectral processing parameters predefined by the Bruker Topspin software. We have predefined two main classes assigned as ‘Schizophrenia’ and ‘Control’ relating to schizophrenia patients and the healthy control group of samples. Using the ascribed methodology, two separate datasets were assembled containing 265 samples (149 of class ‘Schizophrenia’ and 116 of class ‘Control’) gathered in triplicates of the samples, one containing already processed spectra under Bruker Topspin software and another one with reprocessed NMR spectra under GNAT. All other data related to the class and label assignments for the respective samples remained the same in both data sets. Further data pretreatment and modeling were performed in the same way for both data sets. In this way, the potential contribution from random errors should be possible to discriminate. In addition, an independent test data set comprising 32 samples of ‘Schizophrenia’ and 39 samples belonging to the ‘Control’ class, for a total of 71 samples, was also assembled for the purposes of external validation of OPLS-DA models. As in the case of the previous two data sets, the same data pretreatment, including preprocessing, was performed consistently.

#### 4.2.3. Peak Alignment

In contrast to ^1^H-NMR-based metabolomics studies using a reduced data approach [45,46,47], the icoshift peak alignment algorithm allows using the intrinsic spectral resolution of the 500-MHz ^1^H-NMR spectra to extract the information related to the differences in the metabolism between schizophrenia patients and healthy control group. In order to perform chemometrics on NMR spectral data, all peaks in NMR spectra originating from the same chemical surrounding inside the metabolite were aligned. In the first step, shifting the whole spectrum according to the reference signals in the regions 5.12 to 5.19 ppm and 5.20 to 5.35 ppm was performed using 4 iterations, and the target spectrum was chosen from the current data set. For blood samples, an α-D-glucopyranose anomeric doublet, centered at 5.23 ppm, just on the right side of a broad lipid olefinic resonance at 5.27 ppm was chosen [44]. Although this strategy gives satisfying results for the most part of the spectrum, in some regions, mostly where the broad signals overlap among low-intensity metabolite signals, additional alignment was still required. Therefore, in the second step, these spectral areas were separately aligned using SNV (standard normal variate scaling) and first-order derivative interchangeably as the input option for data pretreatment in the icoshift function. Due to the irregular appearance of peaks that could not be aligned in this way (for example, the regions 1.16–1.22 ppm and 3.62–3.69 ppm), some of the individual samples show a potential source of specific variation that would require special attention during the modeling of data.

#### 4.2.4. Data Pretreatment (Preprocessing)

The region between 4.35 and 5.0 ppm was excluded from the data sets before further data pretreatment. Spectra were baselined with a 1st order polynomial baseline function and fitted to predefined regions free of peaks, which were then subtracted from the original spectra. Probabilistic quotient normalization (PQN) [48] was used for normalization. Based on the analysis of skewness and kurtosis, spectral regions below 0.17 ppm and above 8 ppm are excluded. In this way, the variation originating from these areas was significantly reduced. Overall, each data matrix of an initial 32 K was reduced to 15,182 data points in the second dimension.

#### 4.2.5. Cross-Validation (CV)

Next, 7-fold contiguous block CV was adopted for all models, while the size of each block was evaluated in relation to an n-fold CV: N/(3 · s), where s is the number of triplicates, and N is the total number of samples in the data set. For the initial number of samples and 7-fold CV, the size of each block was 13 (patients) · 3 (triplicates) = 39 samples. After removing the outliers, the size of the block was recalculated, keeping the triplicates from the same patients in the same block. In order to preserve the triplicate ordering structure of samples, we have slightly modified the default method of sample assignation to the particular blocks arranged for calibration and validation during the CV. The pre-request for such a step was introducing a separate class variable with assigned triplicate grouping. Randomly shuffling of the samples allowed them to reorder inside the data set with respect to the grouping class variable before each CV step. In this way, we preserved triplicate structure during the shuffling of samples and subsequently assigned them to both calibration and validation blocks of samples.

#### 4.2.6. Transformation and Scaling

The data were examined for natural clusters and outliers by principal component analysis (PCA). Orthogonal partial least squares discriminant analysis (OPLS-DA) was used to classify samples according to clinical and prognostic factors. All spectral variables were mean-centered before PCA analysis. In addition, OPLS-DA was introduced for weight centering. When the group sizes were unequal, the boundary between groups in PLS models were shifted toward the larger group and misclassified many samples. To correct this, the average of the means of the two groups was subtracted from the data matrix, that is, (XA + XB)/2, from the columns [35].

Analyzing the obtained loading plots of mean-centered variables of the data matrix provided straightforward identification of varying metabolite presence in the samples due to their covariance structure and their similarity [28]. However, interpretation can be distorted because some metabolites with apparent covariation in the loadings are not really responsible for the discrimination between different groups or classes. Therefore, regarding multivariate analysis of NMR spectra data matrix, different scaling methods were proposed. Pareto and autoscaling (mean-centering and univariance scaling) methods are most commonly used for weighting all spectral variables according to the square root of standard deviation and standard deviation, respectively. As a result, the data matrix was scaled by multiplying with the inverse of the square root of standard deviation (Pareto), standard deviation (autoscale), and with pooled standard deviation (class centroid centering and scaling) [32] for all variables. However, the resulting loading plots were slightly distorted; therefore, the back-scale projection method proposed by Cloarec et al. [28,31] was used for their explanation in this work.

### 4.3. NMR

NMR experiments were performed on a Bruker Avance III 500 NMR spectrometer equipped with a 5 mm BBI probe head at 25 °C. ^1^H-NMR spectra (1D, 500.26 MHz) were obtained using the presaturation pulse program, Watergate (p3919), with 128 scans, 32K data points and a bandwidth of 12 kHz. The methyl of lactate at 1.33 ppm (3H, ^3^*J* = 7.0 Hz) was used as a referent signal. In addition, we used CPMG (Carr-Purcell-Meiboom-Gill) and T_2_ edited NMR spectra. Additionally, 2D experiments, such as HSQC and TOCSY, were used to confirm the assignments of molecules. The TOCSY experiments used a mlevphpr.2 spin-lock scheme for ^1^H-^1^H transfers. For this experiment, 512 increments with 32 scans were collected. The HSQC experiment was recorded with 256 increments and 120 scans. Together with the 2D experiment assignments and interpretation, the literature and available databases, such as HMDB (Human Metabolome Database), were used to assist in the assignment of molecules.

## 5. Conclusions

Based on serum metabolomics by NMR of a cohort of schizophrenia patients from Serbia, a set of 26 biomarkers for schizophrenia was established. An important discovery is that a great majority of the identified metabolites are equal to the previous reports in Brazil and China on schizophrenia, which opens up a possibility for using these biomarkers as disease markers for diagnostics purposes. Furthermore, four metabolites, aspartate (aspartic acid), lysine, 2-hydroxybutyric acid, and acylglycerols, were identified for the first time in serum samples from this Serbian cohort of patients with schizophrenia based on NMR analyses associated with chemometrics. It is still necessary to discover the universality of the serum biomarkers for schizophrenia independently of geographical and ethnic factors, and for that, a unified analysis of data is necessary.

## Figures and Tables

**Table 1:** Confusion matrix for classification of test data according to PLS-DA model and autoscaling preprocessing.

Actual Class		
	Schizophrenia	Control
Predicted as Schizophrenia	30	0
Predicted as Control	2	39
Predicted as Unassigned	0	0

**Table 2:** Metabolites/biomarkers identified in schizophrenia patients’ serum samples, with spectral data.

No	Metabolites/Biomarkers	TOCSY Correlations (δ_H_, ppm)	2DJ ((δ_H_ (ppm), Multiplicity, J (Hz))	HSQC (δ_H_/δ_c_ (ppm))
1	Lactate/lactic acid	4.10; 1.31	CH_3_: 1.31, d, 6.98; CH: 4.10 q, 7.0	1.32/22.79, 4.098/71.25
2	Threonine	1.31; 3.56; 4.24	CH_3_: 1.32, d, overlapped with lactate; CH: 3.56 d, 5.0; CH_2_: 4.23 dd, 4.9, 6.6, overlapped with acylglycerol	1.34/22.54, 3.55/63.42, 4.24
3	Leucine	0.95; 1.71; 3.71	CH_3_: 0.94, d, 6.24; CH_3_: 0.95, d, 6.24	0.94/23.41, 0.95/24.72, 1.71/42.70, 3.71
4	Valine	0.98; 1.03; 2.27; 3.62	CH_3_: 0.97, d, 7.00; CH_3_: 1.03, d, 7.00; CH: 3.59 d, 4.39	0.97/19.26, 1.02/20.6, 2.27, 3.59/63.27
5	Glutamine	2.12; 2.44; 3.74	CH_2_: 2.12 m; CH_2_: 2.44 m	2.12/29.27, 2.43/33.61, 3.74/57.11
6	Glutamate/glutamic acid	2.05; 2.35; 3.75	CH_2_: 2.04, m and 2.11 m	2.0/29.68, 2.34/36.28, 3.74/57.11
7	Citrate/citric acid	2.51; 2.68	CH_2_: 2.51 d, 16.0; CH_2_: 2.68 d, 16.0	-
8	Aspartate/aspartic acid	2.68; 2.80; 3.88	CH_2_: 2.66, dd, 8.8, 17.5 and 2.80, dd 3.8, 17.4	3.80/54.56
9	Asparagine	2.83; 2.92; 3.96	CH_2_: 2.82 ABX, m, 4.2, 17.0 and 2.93 ABX, m, 7.8, 16.6	-
10	Alanine	1.46; 3.77	CH_3_: 1.46, d, 7.26	3.76/53.21
11	3- Hydroxybutyric acid	1.19; 2.34; 4.12	CH_3_: 1.19 d, 6.4; CH_2_: 2.40, dd, 7.2, 14.4 and 2.29 dd, 6.4, 14.4	-
12	Gamma-aminobutyric acid	1.9; 3.03	CH_2_: 3.04, t, 7.6	-
13	Choline	3.50; 4.05	CH_2_: 4.05 m	4.05/58.35
14	Acylglycerols	4.07; 4.27; 5.20	CH_2_: 4.10 m, 4.23 m overlapped	4.26 and 4.05/64.40; 5.19/71.58
15	Glucose (α + β)	3.40; 3.52; 3.7; 3.75; 5.10; 5.22	CH-4: 3.40 m; CH-2: 3.52 dd, 3.7, 9.7; CH-3: 3.70 m (overlapped); CH_2_-6: 3.75 dd, 5.1, 12.0 and 3.83 m; CH-5: 3.82 m; CH-1: 5.22 d, 3.9	-
16	Arginine	4.07; 4.27; 5.20	3.23 t, 6.6; 1.70, m and 1.64, m	-
17	Lysine	1.70; 1.89; 3.03; 3.74	1.91 m	-
18	2-Hydroxybutyric acid	-	CH_3_: 0.88, t, 7.50; CH_2_: 1.70, m and 1.64, m or arginine	-
19	Isoleucine	-	CH_3_: 0.92, t, 7.4; CH_3_: 0.99, d, 7.0; 3.65 d, 4.04	-
20	Serin	-	CH_2_: 3.97, dd, 3.8, 12.2 and 3.92, dd 5.7, 12.2; CH: 3.82 overlapped	3.95/62.94, 3.81/59.2
21	Mannose	-	CH: 3.55 t, 9.4; CH: 3.79 m; CH: 3.84 dd, 2.2, 4.0; CH: 3.95 m; CH: 5.17, d 1.4	-
22	Glycine	-	CH_2_: 3.54 s	-
23	Glycerol	-	CH_2_: 3.64 and 3.55 m; CH: 3.70 m (overlapped)	3.63 and 3.55/65.31
24	Tyrosine	6.88; 7.18	CH: 3.96, dd, 5.0, 8.1 or phenylalanine; Ar: 6.88 and 7.18	3.95/58.78, Ar: 6.88/118.6, 7.18/133.4
25	Phenylalanine	7.30; 7.36; 7.42	Ar: 7.30 m, 7.37 m, 7.41 m	Ar: 7.31/132.01, 7.40/131.80
26	PABA	6.93; 7.80	-	-

**Table 3:** Metabolites/biomarkers identified in serum samples of Serbian, Brazilian and Chinese patients with schizophrenia, based on NMR analyses.

No	Metabolites/Biomarkers	Serbian Serum Samples	Brazilian Serum Samples	Chines Serum Samples	References
1	Lactate/lactic acid	+	+	+	[9,10,12]
2	Threonine	+	+	+	[9,10]
3	Leucine	+	+	+	[10,12]
4	Valine	+	+	+	[9,10,11,12]
5	Glutamine	+	+	+	[9,10,12]
6	Glutamate/glutamic acid	+	+	−	[9]
7	Citrate/citric acid	+	−	+	[10]
8	Aspartate/aspartic acid	+	−	−	-
9	Asparagine	+	+	+	[9,10,11]
10	Alanine	+	+	+	[9,10,11,12]
11	3-Hydroxybutyric acid	+	−	+	[10]
12	Gamma-aminobutyric acid	+	+	+	[9,11,12]
13	Choline	+	+	+	[10,12]
14	Acylglycerols	+	−	−	-
15	Glucose	+	+	+	[9,10,12]
16	Arginine	+	−	+	[10]
17	Lysine	+	−	−	-
18	2-Hydroxybutyric acid	+	−	−	-
19	Isoleucine	+	+	+	[9,10,12]
20	Serin	+	+	−	[9]
21	Mannose	+	+	−	[9]
22	Glycine	+	+	+	[9,10,12]
23	Glycerol	+	−	+	[10,11]
24	Tyrosine	+	+	+	[10,12]
25	Phenylalanine	+	+	−	[9,12]
26	PABA	+	+	−	[9]
27	Acetylcholine	−	+	−	[12]
28	Mannitol	−	+	−	[9,12]
29	Amygdalin	−	+	−	[9]
30	Lipoamide	−	+	−	[12]
31	Myo-inositol	−	+	+	[10,12]
32	Proline	−	−	+	[10]
33	Acetyl-glycoprotein	−	−	+	[10]
34	Pyruvate	−	−	+	[10,11]
35	Dimethylamine	−	−	+	[10,11]
36	Creatine	−	+	+	[10,12]
37	Taurine	−	−	+	[10,11]
38	3-Methylhistidine	−	−	+	[10]
39	Hypotaurine	−	−	+	[11]
40	Malonate	−	−	+	[11]
41	Guanidinoacetic acid	−	−	+	[11]
42	Propylene glycol	−	−	+	[11]
43	Threitol	−	−	+	[11]
44	Acetoacetate	−	−	+	[11]
45	Methymalonic acid	−	−	+	[11]
46	Malic acid	−	−	+	[11]
47	N-Acetylglycine	−	−	+	[11]
48	Dimethylglycine	−	−	+	[11]
49	Betaine	−	−	+	[11]
50	Arabitol	−	−	+	[11]
51	Xylitol	−	−	+	[11]
52	Phosphocholine	−	+	−	[11,12]
53	2-Methylglutaric acid	−	−	+	[11]
54	Fructose	−	−	+	[11]
55	D-Gluconic acid	−	−	+	[11]
56	Galactitol	−	−	+	[11]
57	Homovanillic acid	−	−	+	[11]
58	Methylamine	−	−	+	[11]
59	6-Hydroxydopamine	−	+	−	[12]
60	Isovaleryl carnitine	−	+	−	[12]
61	Pantothenate	−	+	−	[9,12]
62	Guanine	−	+	−	[9]
63	3-methyl-2-oxobutunoic acid	−	+	−	[9]

## Data Availability

Data available on request due to restrictions eg privacy or ethical. The data presented in this study are available on request from the corresponding author. The data are not publicly available due to privacy and ethical restrictions.

## Supplementary Materials

The following supporting information can be downloaded at: https://www.mdpi.com/article/10.3390/metabo12080707/s1, Figure S1: (a) ^1^H-NMR spectra processed under Bruker Topspin software. (b) Same spectra with phase of 0th order, corrected under GNAT program; Figure S2: (a) Results for skewness and kurtosis in area of spectra with significant discrepancies from regular value for normal distribution (0 for skewness and 3 for kurtosis); (b) NMR spectra in the region, where spectra colored in red indicate potential outliers. (c) Results for skewness and kurtosis in area of spectra after removal of samples identified as potential outliers. The horizontal dotted lines on both Figures are passing/going through the given values for skewness and kurtosis (0 and 3, r) ef any univariate normal distribution. (d) NMR spectra in the corresponding region after removal of spectra colored in red (b); Figure S3: (a) Mean-centered NMR spectra referring to the samples marked with ellipses in Figure 1a, in red color; (b) Several NMR spectra of samples that originate from the central part of the scores plot presented in Figure 1a are given in blue; Figure S4: (a) Scores plot of PCA model presented in PC 1 vs. PC2 components. Scaling and centering were accomplished with autoscaling; (b) Corresponding loading plot of PC 1 component.; Figure S5: (a) Y Predicted for the class ‘Schizophrenia’ and (b) Y Predicted for the class ‘Control’ from external test data set, using autoscaling; Figure S6: Fractional Y-variance captured for self-prediction (calibration) and cross-validation versus the correlation of the permuted Y-block to the original Y-block for 4 LV component models of (a) OPLS-DA model using autoscaling and (b) OPLS-DA model using class centroid centering and scaling.
