# Supplementary material for: NMR Metabolomics in Serum Fingerprinting of Schizophrenia Patients in a Serbian Cohort
Source: Metabolites. 2022 Jul 29;12(8):707. doi: 10.3390/metabo12080707 (PMC9416612; doi:10.3390/metabo12080707)
Supplement: Supplementary file 1 [file metabolites-12-00707-s001.zip › metabolites-1812008-supplementary.pdf]

NMR metabolomics in serum fingerprinting of Schizophrenia Patients in the Serbian cohort

Katarina Simić[1], Nina Todorović[1], Snežana Trifunović [2], Zoran Miladinović [3], Aleksandra Gavrilović[4], Silvana Jovanović [4], Nataša Avramović [5], Dejan Gođevac[1], Ljubodrag Vujisić [2], Vele Tešević [2], Ljubica Tasić [6] and Boris Mandić [2,*]

1 Institute of Chemistry, Technology and Metallurgy, National Institute, University of Belgrade, Studentski trg 12-16, 11000 Belgrade, Serbia; katarina.simic@ihtm.bg.ac.rs, ninat@chem.bg.ac.rs, dgodjev@chem.bg.ac.rs
2 University of Belgrade - Faculty of Chemistry, Studentski trg 12-16, 11000 Belgrade, Serbia; snezanat@chem.bg.ac.rs, ljubaw@chem.bg.ac.rs, vtesevic@chem.bg.ac.rs, borism@chem.bg.ac.rs
3 Institute of General and Physical Chemistry, Studentski trg 12-16, 11158 Belgrade, Serbia; zmiladinovic@iofh.bg.ac.rs
4 Special hospital for psychiatric diseases "Kovin", Cara Lazara 253, 26220 Kovin, Serbia; gavrilovicaleksandra74@gmail.com, silvana.jovanovic555@gmail.com
5 Institute of Medical Chemistry, Faculty of Medicine, University of Belgrade, 11000 Belgrade, Serbia; natasa.avramovic@med.bg.ac.rs
6 Institute of Chemistry, Organic Chemistry Department, State University of Campinas, Campinas, SP, Brazil; ljubica@unicamp.br

* Correspondence: borism@chem.bg.ac.rs; Tel.: +381 11 2630477

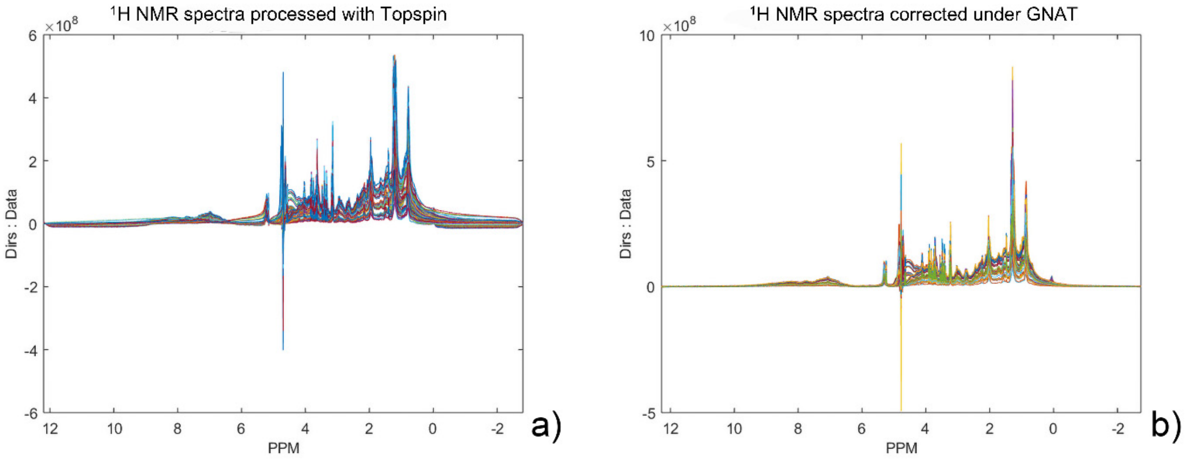

Figure S1. (a) ^{1}H-NMR spectra, processed using the Bruker Topspin software. **(b)** The same spectra with the phase of 0th order, corrected under the GNAT program.

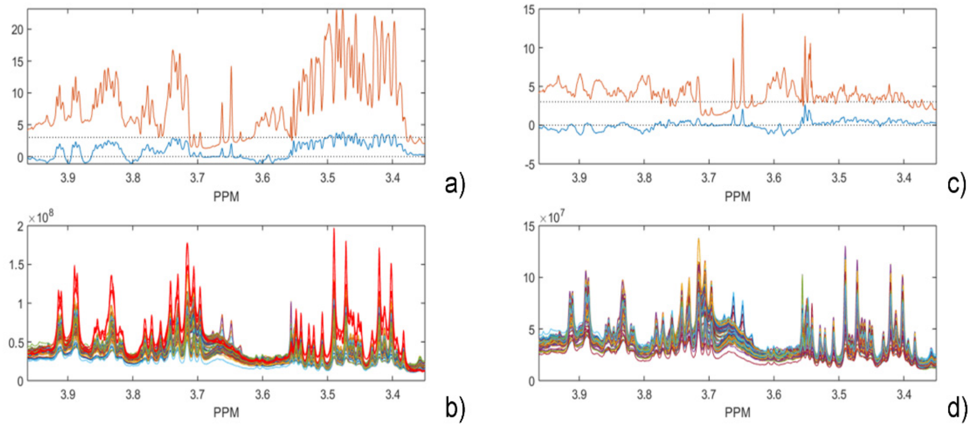

Figure S2. a) Results for skewness and kurtosis in the area of spectra with significant discrepancies from common value for normal distribution (0 for skewness and 3 for kurtosis); **b)** NMR spectra in the region, where spectra colored in red indicate potential outliers. **c)** Results for skewness and kurtosis in the area of spectra after removing the samples identified as potential outliers. The horizontal dotted lines on both Figures are passing/going through the given values for skewness and kurtosis (0 and 3, r) of any univariate normal distribution. **d)** NMR spectra in the corresponding region after removing the spectra colored in red (b).

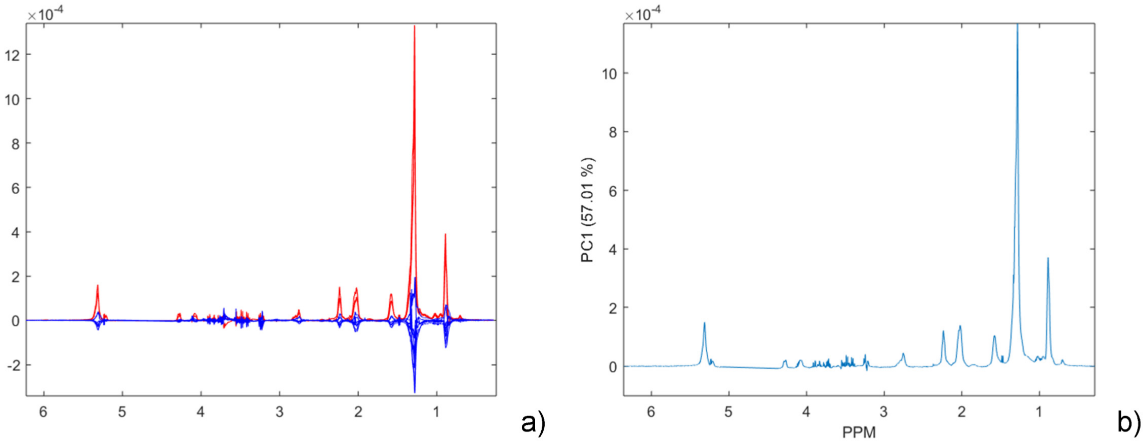

Figure S3. a) Mean-centered NMR spectra referring to the samples marked with ellipses in Figure 1a, in red color;

b) Several NMR spectra of samples that originate from the central part of the score plot presented in Figure 1a are given in blue.

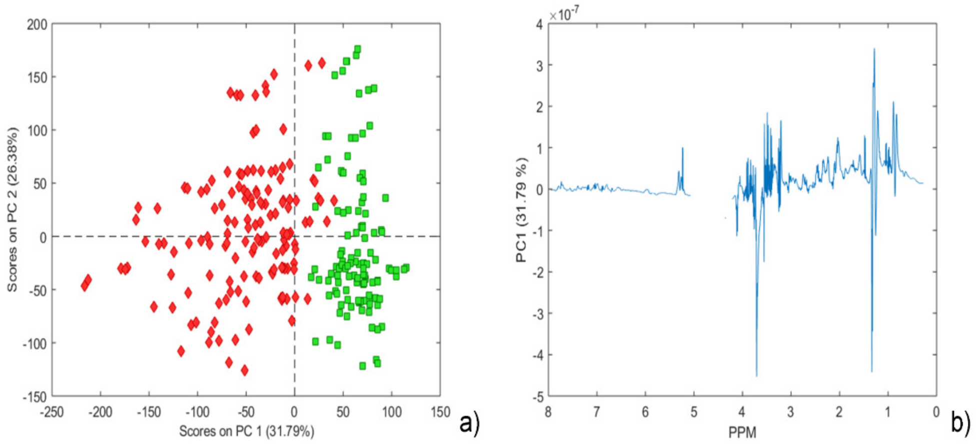

Figure S4. a) Score plot of PCA model presented in PC 1 vs. PC2 components. Scaling and centering were accomplished with autoscaling; **b)** Corresponding loading plot of PC 1 component.

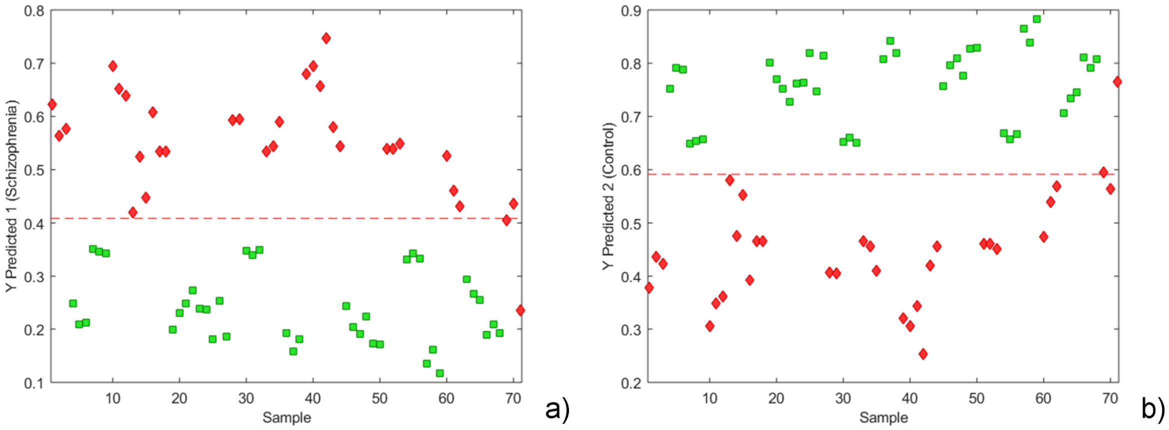

Figure S5. a) Y Predicted for the class 'Schizophrenia' and **b)** Y Predicted for the class 'Control' from external test data set, using autoscaling.

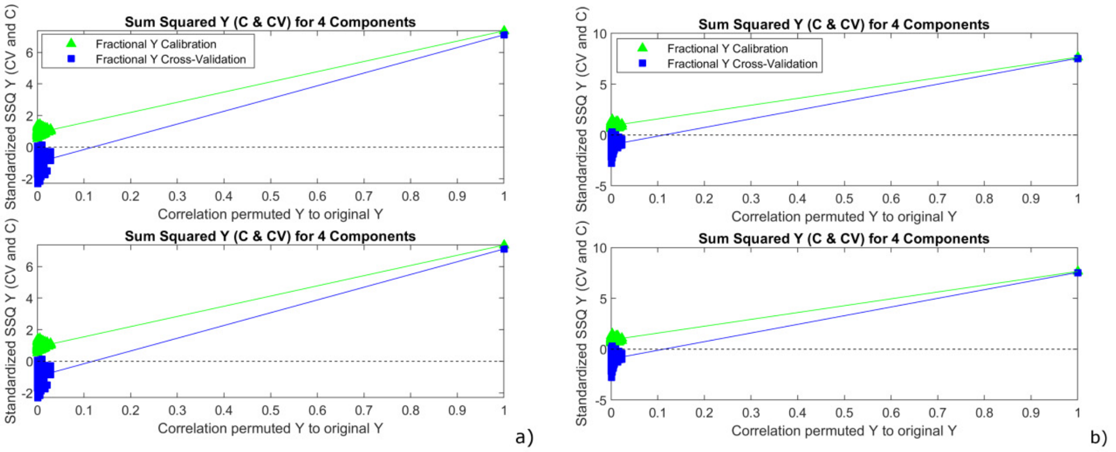

Figure S6. Fractional Y-variance captured for self-prediction (calibration) and cross-validation versus the correlation of the permuted Y-block to the original Y-block for 4 LV component models of **a)** OPLS-DA model using autoscaling and **b)** OPLS-DA model using class centroid centering and scaling.
